# Research Progress on Natural Diterpenoids in Reversing Multidrug Resistance

**DOI:** 10.3389/fphar.2022.815603

**Published:** 2022-03-28

**Authors:** Zhuo-fen Deng, Irina Bakunina, Hua Yu, Jaehong Han, Alexander Dömling, Maria-José U Ferreira, Jian-ye Zhang

**Affiliations:** ^1^ Key Laboratory of Molecular Target & Clinical Pharmacology, The State & NMPA Key Laboratory of Respiratory Disease, School of Pharmaceutical Sciences and the Fifth Affiliated Hospital, Guangzhou Medical University, Guangzhou, China; ^2^ G.B. Elyakov Pacific Institute of Bioorganic Chemistry, Far Eastern Branch, Russian Academy of Sciences, Vladivostok, Russia; ^3^ Institute of Chinese Medical Sciences, State Key Laboratory of Quality Research in Chinese Medicine, University of Macau, Macao SAR, China; ^4^ Metalloenzyme Research Group and Department of Plant Science and Technology, Chung-Ang University, Anseong, Korea; ^5^ Department of Pharmacy, University of Groningen, Groningen, Netherlands; ^6^ Research Institute for Medicines (iMed.ULisboa), Faculty of Pharmacy, Universidade de Lisboa, Lisboa, Portugal

**Keywords:** cancer, chemotherapy, multidrug resistance, natural products, diterpenoids

## Abstract

Multidrug resistance (MDR) is one of the main impediments in successful chemotherapy in cancer treatment. Overexpression of ATP-binding cassette (ABC) transporter proteins is one of the most important mechanisms of MDR. Natural products have their unique advantages in reversing MDR, among which diterpenoids have attracted great attention of the researchers around the world. This review article summarizes and discusses the research progress on diterpenoids in reversing MDR.

## Introduction

Cancer, one of major public health problems, imposes a serious challenge to the survival of human beings worldwide (Wu et al., 2019). Although there are several different cancer treatment modalities, chemotherapy is still one of the main approaches of cancer therapy (Bukowski et al., 2020). However, the development of chemoresistance especially multidrug resistance (MDR) has greatly restricted the effectiveness of drugs for cancer management, which can result in treatment failure (Holohan et al., 2013). MDR refers to the resistance of cancer cells to various chemotherapy drugs with different structures and mechanisms. Therefore, there is a need to clarify the mechanisms of MDR and seek some effective reversal strategies.

At present, many MDR reversal agents have been developed to overcome MDR. Natural products, characterized for having high binding ability to various biological targets, and frequently low toxicity, might be crucial for overcoming MDR (Guo et al., 2017). A significant number of studies have shown that natural products possessed the potential to reverse MDR (Kumar and Jaitak, 2019). Diterpenoids, an important group of bioactive compounds in natural products, have been playing an important role in drug discovery. In recent years, it was found that some diterpenoids, mostly macrocyclic diterpenes, were able to reverse MDR in cancer cells (Molnar et al., 2006; Kumar and Jaitak, 2019). This review discusses the research progress on diterpenoids in reversing MDR.

## Multidrug Resistance

### ATP-Binding Cassette Transporter-Mediated Multidrug Resistance

MDR of cancer cells is associated with various mechanisms (Bukowski et al., 2020). Of all mechanisms, increased drug-efflux of structurally different anticancer drugs, mediated by ABC-transporter proteins is a common one (Borst and Elferink, 2002). In the 1970s, ABCB1, a member of ABC transporters, was first discovered (Juliano and Ling, 1976). To date, other ABC transporters have been found, such as ABCC1, ABCC2 and ABCG2, which are associated with MDR (Takano et al., 2006; Lu et al., 2015). Besides the involvement in MDR, transmembrane transport of endogenous or exogenous molecules is one of the main physiological functions of ABC transporters (Wang JQ et al., 2021). They possess the function of energy dependent “drug-pump”.

### ATP-Binding Cassette Subfamily B Member 1

ABCB1 is one of the research hotspots in ABC transporter family because its expression is up-regulated in many drug-resistant and refractory tumors (Kadioglu et al., 2016). Studies have shown that the expression of ABCB1 is regulated by various signaling pathways, such as nuclear factor κB (NF-κB) (Sun et al., 2012), mitogen-activated protein kinases (MAPK) (Luo et al., 2016) and phosphoinositide-3-kinase–protein kinase B (PI3K-PKB) (Dong et al., 2017). Therefore, by clarifying these signaling pathways, it may help exploring targets for reversing MDR.

At present, four generations of ABCB1 inhibitors have been developed. The first-generation reversal inhibitors include calcium channel blockers, immunosuppressants, protein kinase C inhibitors and so on. These inhibitors have low affinity and high toxicity (Shen et al., 2008). The second-generation inhibitors were obtained by improving the first-generation inhibitors, including dexverapamil, biricodar. Compared with the first-generation inhibitors, they have stronger affinity for ABCB1, less toxicity and better effect (Thomas and Coley, 2003). The third-generation of ABCB1 inhibitors, such as tariquidar and zosuquidar, were much more effective than the first-generation and second-generation inhibitors (Martin et al., 1999). However, further development of the third-generation ABCB1 inhibitors was limited by some unexpected side effects in clinical trials (Chen et al., 2017). The fourth-generation ABCB1 inhibitors include 1) peptidomimetics, 2) compounds isolated from natural sources and their derivatives, and 3) dual ligands (compounds capable of inhibiting ABCB1 and another mediator of MDR) (Dong et al., 2020). Many of them possess both antitumoral and MDR reversing activities. Nevertheless, most of fourth-generation ABCB1 inhibitors have been evaluated in cancer cells *in vitro*, and their efficacy and safety *in vivo* have not been determined.

### Diterpenoids as Multidrug Resistance Reversal Agents

Diterpenoids (C20), one of the largest groups of natural products, are derived from four C5 isoprene units. The main skeleton types of diterpenoids include, among others, kaurenes, clerodanes, abietanes and labdanes. Lathyranes and jatrophanes are macrocyclic diterpenes characteristic of *Euphorbia* genus (Euphorbiaceae family), from which many compounds showed significant MDR reversing activity. Some other diterpenoids from *Euphorbia* species were also reported as MDR reversers, such as ingenanes, segetanes, and jatropholanes. Some diterpenes from other plants, such as *Pseudolarix*, *Taxus*, *Briareum*, *Sindora* species and so on, also possess certain reversal activity.

### Jatrophanes

Six new jatrophane-type diterpenoids and three known diterpenes were isolated from the whole undried plants of *Euphorbia esula* (Table 1), most of which were able to enhance the Rhodamine 123 (Rh123) accumulation in human *ABCB1*-transfected L5178Y mouse T-lymphoma cells, overexpressing ABCB1 (Vasas et al., 2011). Rh123 accumulation assay is commonly used to characterize potential ABCB1 inhibitors (Jouan et al., 2016). According to the experimental results, compounds **1** and **2** were the most powerful inhibitors of ABCB1 efflux-pump activity, whose efficacy was 2–5 times higher than that of the standard modulator verapamil [Fluorescence activity ratio (FAR) = 52.5 at 40 μg/ml and 119.9 at 40 μg/ml]. The FAR was calculated on the basis of the measured fluorescence values via the following equation: FAR = $\frac{\mathrm{MDR}\;\mathrm{treated}/\mathrm{MDR}\;\mathrm{control}}{\mathrm{parental}\;\mathrm{treated}/\mathrm{parental}\;\mathrm{control}}$ (Vasas et al., 2011).

**TABLE 1:** Jatrophanes. 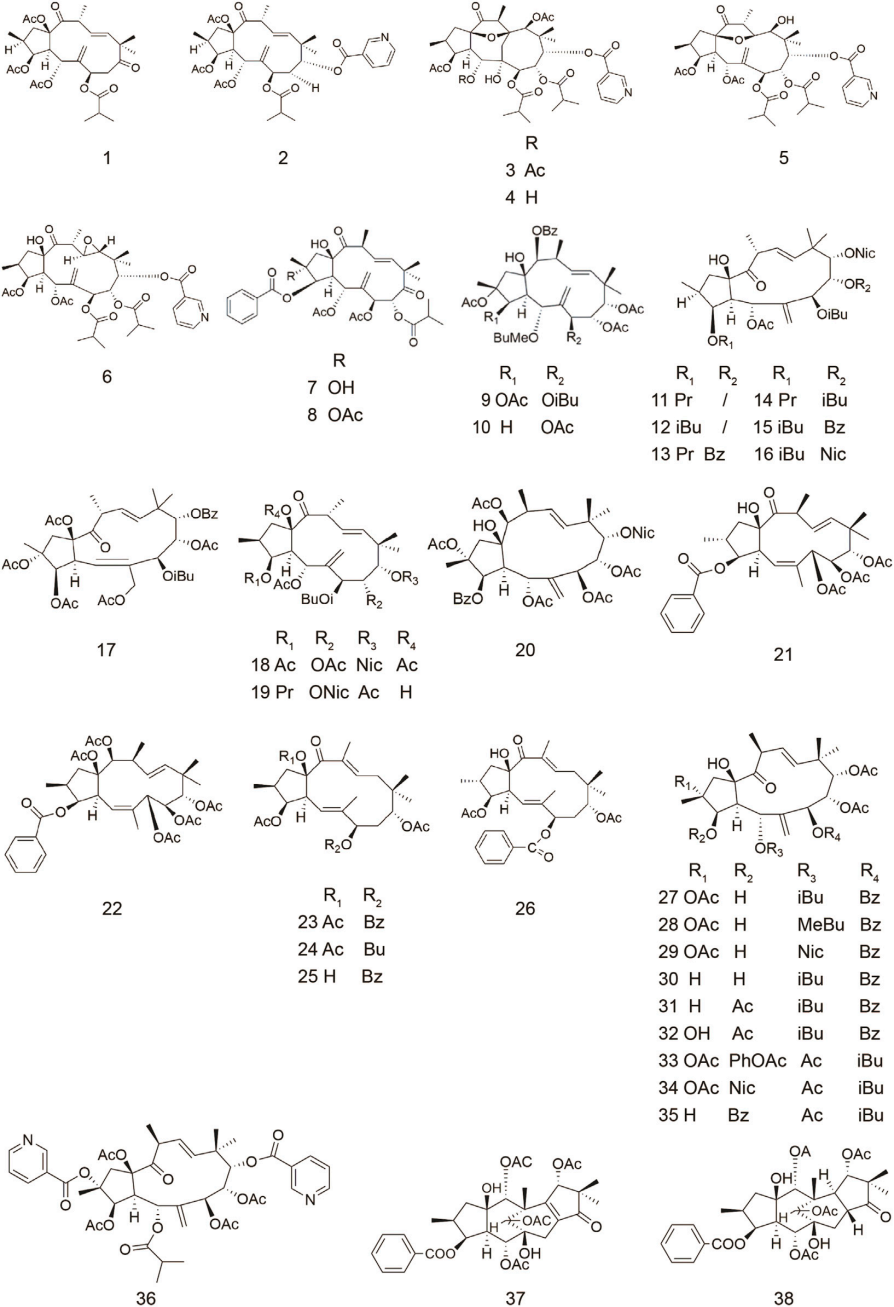

Compound	Name	Plant	Ref
**1**	esulatin J	*E. esula*	Vasas et al. (2011)
**2**	esulatin M	*E. esula*	Vasas et al. (2011)
		*E. welwitschii*	Reis et al. (2016)
**3**	euphowelwitschine A	*E. welwitschii*	Reis et al. (2016)
**4**	euphowelwitschine B	*E. welwitschii*	Reis et al. (2016)
**5**	welwitschene	*E. welwitschii*	Reis et al. (2016)
**6**	epoxywelwitschene	*E. welwitschii*	Reis et al. (2016)
**7**	euphoglomeruphane K	*E.glomerulans*	Hasan et al. (2019)
**8**	euphoglomeruphane L	*E.glomerulans*	Hasan et al. (2019)
**9**	euphosorophane A	*E.sororia*	Hu et al. (2018)
**10**	euphosorophane I	*E.sororia*	Yang et al. (2021)
**11**	euphodendrophane A	*E. dendroides*	Aljancic et al. (2011)
		*E. nicaeensis*	
			Krstic et al. (2018)
**12**	euphodendrophane B	*E. dendroides*	Aljancic et al. (2011)
		*E. nicaeensis*	
			Krstic et al. (2018)
**13**	euphodendrophane H	*E. dendroides*	Jadranin et al. (2013)
**14**	euphodendrophane J	*E. dendroides*	Jadranin et al. (2013)
**15**	euphodendrophane K	*E. dendroides*	Jadranin et al. (2013)
**16**	euphodendrophane L	*E. dendroides*	Jadranin et al. (2013)
**17**	euphodendrophane S	*E. dendroides*	Jadranin et al. (2013)
**18**	nicaeenin F	*E. nicaeensis*	Krstic et al. (2018)
**19**	nicaeenin G	*E. nicaeensis*	Krstic et al. (2018)
**20**	pepluanin A	*E. peplus*	Corea et al. (2004)
**21**	euphomelliferine	*E. mellifera*	Valente et al. (2012)
**22**	euphomelliferene A	*E. mellifera*	Valente et al. (2012)
**23**	pubescene A	*E. pubescens*	Valente et al. (2004)
			Ferreira et al. (2005)
**24**	pubescene B	*E. pubescens*	Valente et al. (2004)
			Ferreira et al. (2005)
**25**	pubescene C	*E. pubescens*	Valente et al. (2004)
			Ferreira et al. (2005)
**26**	pubescene D	*E. pubescens*	Valente et al. (2004)
			Ferreira et al. (2005)
**27**	euphodendroidin A	*E. dendroides*	Corea et al. (2003)
**28**	euphodendroidin B	*E. dendroides*	Corea et al. (2003)
**29**	euphodendroidin C	*E. dendroides*	Corea et al. (2003)
**30**	euphodendroidin D	*E. dendroides*	Corea et al. (2003)
**31**	euphodendroidin E	*E. dendroides*	Corea et al. (2003)
**32**	euphodendroidin F	*E. dendroides*	Corea et al. (2003)
**33**	Jatrophane diterpene	*E. dendroides*	Corea et al. (2003)
**34**	euphodendroidin G	*E. dendroides*	Corea et al. (2003)
**35**	euphodendroidin H	*E. dendroides*	Corea et al. (2003)
**36**	euphodendroidin I	*E. dendroides*	Corea et al. (2003)
**37**	euphoportlandol A	*E. portlandica*	Madureira et al. (2006)
**38**	euphoportlandol B	*E. portlandica*	Madureira et al. (2006)

Compounds **2**-**6** were isolated from *Euphorbia welwitschii* (Table 1). The property of interaction of compound **6** with ABCB1 was studied by ATPase assay (Reis et al., 2016). Two complementary assays compose the ATPase experiment (activation assay to test the effect on the basal ATPase activity, inhibition assay to test the effect on drug-stimulated ATPase activity). Maintaining the efflux function of ABCB1 requires energy generated by ATP hydrolysis, which requires ATPase (Szollosi et al., 2018). The ATPase activity of ABCB1 is one of the most attractive targets for the design of inhibitors (Mollazadeh et al., 2018). The measurement of catalytic activity is a means of investigating candidate regulators as substrates or inhibitors, and inhibited the verapamil-stimulated ATPase activity, being a complete inhibition attained at 50 and 100 μM. The effects of compound **6** on the ATPase activity of ABCB1 showed it to interact with the transporter and to be able to reduce the transport of a second substrate. The Rh123 efflux assay results also showed that all these compounds were able to inhibit the efflux activity of ABCB1 at 20 µM. Their efficacy was 2–3 times higher than that of the positive control verapamil in a mouse T-lymphoma *ABCB1*-transfected cell model (FAR = 12.5 at 20 μM) (Reis et al., 2016).

Seventeen new jatrophane diterpenoids and five known were isolated from the whole plant of *Euphorbia esula* (Table 1). Their reversal fold (RF) values on MCF-7/ADR cells overexpressing ABCB1 (MCF-7 cell line with adriamycin resistance) ranged from 2.3 to 12.9 at 10 μΜ. The methods used to assay MDR-reversal activity mainly include RF and FAR values. RF value can be calculated by the MTT method, which can reveal reversal activities and cytotoxicity of compounds. The Rh123 efflux assay is to determine whether compounds have an effect on the function of ABCB1 transport substrate, from which FAR value is calculated. Compared to RF value, FAR value can give a direct quantitative assessment whether compounds modulate the efflux mediated by ABCB1. Their reversal fold values on MCF-7/ADR cells overexpressing ABCB1 ranged from 2.3 to 12.9 at 10 μΜ. Among them, the MDR reversal activities of compounds **7** and **8** were as good as that of verapamil (RF = 13.7), with RF values of 12.9 and 12.3 at 10 μM, respectively (Hasan et al., 2019).

Five new jatrophane diterpenoids were isolated from the fructus of *Euphorbia sororia* (Table 1). Among them, the most effective compound was compound **9**. Compound **9** showed reversal potency with RF values of 36.82, 20.59 at a concentration of 10.0 mM in the MCF-7/ADR cells. The advantages of compound **9** included high potency (EC_50_ = 92.68 ± 18.28 nM) in overcoming ABCB1-mediated MDR to adriamycin (ADR), stimulating ABCB1-ATPase activity in a concentration-dependent manner, and potency in reversing resistance to other cross-resistant chemotherapeutic agents, such as ADR and vincristine (VCR), and inhibition of ABCB1-mediated Rh123 efflux function in MCF-7/ADR cells. In addition, it did not downregulate the expression of ABCB1 in the MCF-7/ADR cells. The Dixon plot analysis indicated that compound **9** was competitive inhibitor of ABCB1-mediated ADR transport, which was in agreement with the Lineweaver-Burk analysis (Hu et al., 2018).

Also, eight new and fourteen known were isolated from the fructus of *Euphorbia sororia*. Among them, fourteen compounds showed lower cytotoxicity and promising ability to reverse MDR, compared to verapamil, which was used as positive control. Within these jatrophanes, compound **10** appeared to be the most powerful ABCB1 inhibitor (EC_50_ = 1.82 μM). Fluorescence microscopy showed that compound **10** was able to enhance the Rh123 accumulation of multidrug-resistant cells in a dose-dependent manner. Further studies showed that compound **10** stimulated ABCB1-ATPase activity in a concentration-dependent manner instead of down regulating ABCB1 expression and mRNA levels (Yang et al., 2021).

Seven new diterpenoids were isolated from *Euphorbia dendroides* and were investigated for the biological activities on the MDR cell line NCI-H460/R. These compounds included six new jatrophanes, among which two compounds (**11** and **12**, Table 1) exerted high potency in overcoming ABCB1-mediated MDR (FAR = 3.0 to 3.2 at 20 μM). The results suggested that they had the potential to reverse the drug resistance of ADR and paclitaxel (TAX) in the MDR cancer cell line. Notablely, it was showed for the first time that a synergistic effect existed between the TAX and jatrophanes (Aljancic et al., 2011).

Some jatrophane diterpenoids, including compounds **11** and **12**, were isolated from the latex of *Euphorbia nicaeensis*, together with seven previously undescribed jatrophanes (Table 1), among which compounds **18** and **19** were the most active compounds (FAR = 4.52 and 5.02 at 5 μΜ on non-small cell lung carcinoma NCI-H460/R, FAR = 5.89 and 4.39 on colorectal carcinoma DLD1-TxR) (Krstic et al., 2018).

Some new diterpenes were isolated from the whole plant of *Euphorbia peplus *and their inhibitory activity to ABCB1 was investigated in ABCB1-overexpressing K562/R7 human leukemic cells (Table 1). The results showed that compound **20** was the most active inhibitor, whose efficiency was at least two-fold higher than the conventional modulator, which was taken here as reference (100%) (cyclosporin A, 5 µM). The study on structure activity relationship (SAR) showed the importance of substitution on medium-size rings (carbons 8, 9, 14 and 15) (Corea et al., 2004).

Five jatrophane diterpenes, including three new compouds, were isolated from *Euphorbia mellifera* (Table 1). Compounds **21** and **22** exhibited significant activity on multidrug-resistant mouse lymphoma cells and on human colon adenocarcinoma cells in a dose-dependent manner. (FAR = 12.1, 23.1 at 20 μM, and FAR = 72.9, 82.2 at 60 μM respectively, on MDR mouse lymphoma cells; FAR = 5.1 and 5.5 at 20 μM on human colon adenocarcinoma cells) (Valente et al., 2012).

Four jatrophane diterpenes were isolated from *Euphorbia pubescens* (compounds **23**–**26,** Table 1) (Valente et al., 2004). The anti-MDR activities of these compounds were investigated on mouse lymphoma cells. All the compounds displayed a significant effect on inhibiting ABCB1 efflux-pump activity compared with that of the positive control verapamil (FAR = 21.28 at 20 μM) (Ferreira et al., 2005).

Ten jatrophane diterpenes were isolated from *Euphorbia dendroides* (Table 1). A SAR study showed the general effect of lipophilicity on activity, and also emphasized the correlation of substitution patterns at positions 2, 3 and 5, indicating that the fragment was involved in binding. Among all these compounds, compound **30** was the most active inhibitor in ABCB1-overexpressing human K562/R7 leukemic cells, which was almost two-fold more efficient (183 ± 17% at 5 µM) than cyclosporin A, which was taken here as reference (100%) (Corea et al., 2003).

Compounds **37** and **38** are rearranged jatrophane diterpenoids of the segetane group that were isolated from *Euphorbia portlandica* (Table 1). Their biological activity was investigated against MDR in human *ABCB1*-gene transfected mouse lymphoma cells. The result showed that both compounds were effective (FAR = 40.3 and 30.7 at 40 μg/ml, respectively). When comparing the results with those found for macrocyclic jatrophanes, the authors concluded that these rearranged derivatives were less active. Thus, according to the authors, the macrocycle scaffold of these diterpenes and its substitution pattern seem to play an important role in reversing ABCB1-mediated MDR (Madureira et al., 2006).

Some studies on a structurally heterogeneous set of jatrophane polyesters revealed the positive effect of overall lipophilicity on ABCB1 binding and suggested the importance of the oxygen substituent at C-9 (Hohmann et al., 2002). A study showed that the saturated five membered ring had an important effect on the activity (Zhu et al., 2016).

### Lathyranes

Two highly modified lathyrane diterpenoids were isolated from the leaves and twigs of *Jatropha gossypiifolia* (Table 2). The ability of both compounds as MDR modulators was assessed on ADR-resistant HepG2/ADR and HCT-15/5-FU cell lines. The results suggested that only compound **40** showed decent activity, with RF values of 3.3 and 5.8 at 10 μM, respectively on the two cell lines, compared to verapamil (RF = 6.2). In addition, compound **40** had no intrinsic cytotoxicity to both of the MDR cell lines (Li et al., 2020).

**TABLE 2:** Lathyranes. 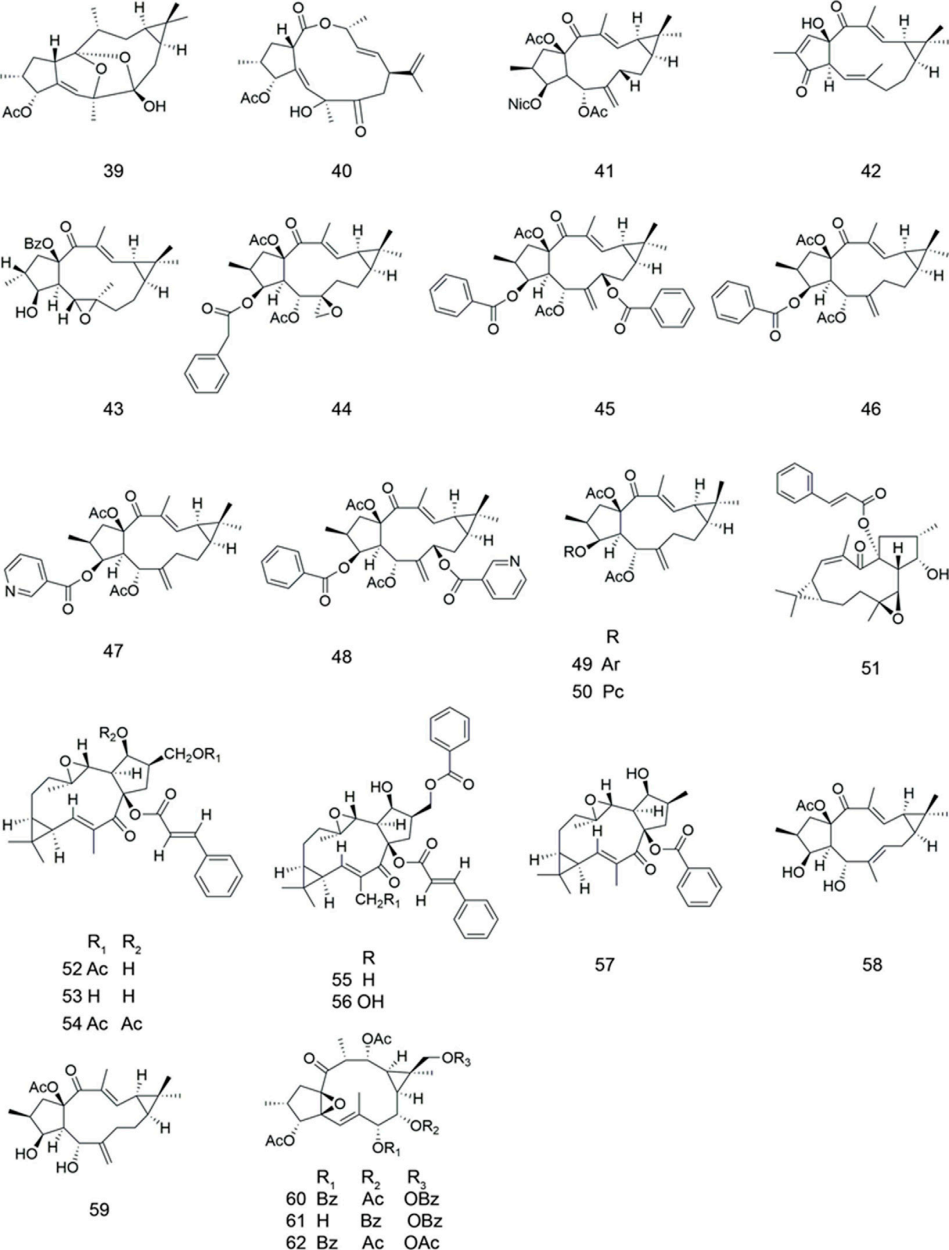

Compound	Name	Plant	Ref
**39**	jatrofoliane A	*J. gossypiifolia*	Li et al. (2020)
**40**	jatrofoliane B	*J. gossypiifolia*	Li et al. (2020)
**41**	5, 15-di-O-acetoxy-3-nicotinoyllathyol-6,12-diene-14-one	*E. lathyris*	Yang et al. (2020)
**42**	macrorilathyrone A	*E. macrorrhiza*	Gao et al. (2016)
**43**	macrorilathyrone B	*E. macrorrhiza*	Gao et al. (2016)
**44**	euphorbia factor L1	*E. lathyris*	Zhang et al. (2011)
			Zhang et al. (2013)
**45**	euphorbia factor L2	*E. lathyris*	Teng et al. (2018)
**46**	euphorbia factor L3	*E. lathyris*	Teng et al. (2018)
**47**	euphorbia factor L8	*E. lathyris*	Teng et al. (2018)
**48**	euphorbia factor L9	*E. lathyris*	Teng et al. (2018)
**49**	euphoboetirane A	*E. boetica*	Neto et al. (2019)
**50**	euphoboetirane B	*E. boetica*	Neto et al. (2019)
**51**	EM-E-11–4	*E. micractina*	Liu et al. (2015)
			Liu et al. (2020)
**52**	latilagascene A	*E. lagascae*	Duarte et al. (2006)
			Duarte et al. (2007)
**53**	latilagascene B	*E. lagascae*	Duarte et al. (2006)
			Duarte et al. (2007)
**54**	latilagascene C	*E. lagascae*	Duarte et al. (2006)
			Duarte et al. (2007)
**55**	latilagascene D	*E. lagascae*	Duarte et al. (2006)
			Duarte et al. (2007)
**56**	latilagascene E	*E. lagascae*	Duarte et al. (2007)
**57**	latilagascene F	*E. lagascae*	Duarte et al. (2007)
**58**	piscatoriol A	*E. piscatoria*	Reis et al. (2014)
**59**	piscatoriol B	*E. piscatoria*	Reis et al. (2014)
**60**	euphornan K	*E. marginata*	Zhang et al. (2020)
**61**	euphornan N	*E. marginata*	Zhang et al. (2020)
**62**	euphornan R	*E. marginata*	Zhang et al. (2020)

Four new lathyrol-type diterpenoids and some known diterpenoids were isolated from *Euphorbia Lathyris* (Table 2). All the compounds were evaluated for MDR reversing activity against HepG2/ADR cells. Most of them were able to reverse MDR, with RF values of 10.05–448.39 at 20 μM. Among them, compound **41** showed the best activity. To investigate the mechanism of reversing MDR of lathyrane diterpenes, Yang et al. examined the effect of compound **41** on the cell viability of HepG2/ADR cells and the ADR accumulation of HepG2/ADR in the presence of the compound at 20 µM. The results showed that this compound with the best MDR reversing activity had low cytotoxicity and was able to promote ADR accumulation in HepG2/ADR cells in time-dependent model (Yang et al., 2020).

Twenty diterpenoids were isolated from *Euphorbia macrorrhiza*, including two lathyranes, namely compounds **42** and **43** (Table 2). Among them, compound **43** showed significant inhibitory activity on ABCB1-mediated drug efflux in KBv200 cell line (RF = 43.63) The inhibitory effect of compound **43** on ABCB1-mediated drug efflux was further tested at several concentrations by Rh123 accumulation assay. Compound **43** exhibited significant effect in increasing the intracellular accumulation of Rh123 (FAR = 2.12 at 30 μM) when compared with the postive control verapamil (FAR = 1.63 at 10 μM) (Gao et al., 2016).

Compound **44** was isolated from Caper *Euphorbia* seed (seeds of *Euphorbia lathyris*) (Table 2). For the first time, researchers showed that compound **44** enhanced the sensitivity of established ABCB1 substrates and increased accumulation of ADR and Rh123 in ABCB1-mediated MDR KBv200 and MCF-7/ADR cells. In the meantime, compound **44** did not downregulate the expression of ABCB1 either in protein or mRNA level (Zhang et al., 2011). A further study was conducted on reversal activities of compound **44** against ABCB1-mediated MDR and apoptosis sensitization in K562/ADR cells. The results showed that the combination of compound **44** and ABCB1 substrate chemotherapeutic drugs may help to overcome MDR. The mitochondrial pathway was involved in the apoptosis sensitization by compound **44** (Zhang et al., 2013). The cytotoxicity of compounds **44**–**48** was evaluated against A549, MDA-MB-231, KB, and MCF-7 cancer cell lines and the KB-VIN MDR cancer cell line. Compound **45** exhibited selectivity against KB-VIN and compound **48** showed the strongest cytotoxicity (Teng et al., 2018).

Compounds **49** and **50** were isolated from *Euphorbia boetica* (Table 2). The activity of reversing MDR was evaluated using a combination of transport and chemosensitivity assays in L5178Y-MDR and Colo320 cell models. The results confirmed the importance of macrocyclic lathyrane diterpenes as effective lead compounds for reversing MDR (Neto et al., 2019).

Compound **51**, isolated from *Euphorbia micractina*, was found to remarkably increase TAX uptake in Caco-2 cells overexpressing ABCB1 (Table 2). The results showed that compound **51** was an effective potential drug to reverse ABCB1-mediated MDR by inhibiting ABCB1 transport function and increasing the intracellular concentration of TAX (Liu et al., 2015). Further study has showed that compound **51** could reverse βIII-tubulin and ABCB1-mediated TAX resistance in tumor cells. Most notably, it was showed for the first time that a small molecule natural product could specifically inhibit the expression of βIII-tubulin (Liu et al., 2020). Some research showed that overexpression of βIII-tubulin might contribute to chemotherapy resistance (Katsetos et al., 2003; Katsetos and Draber, 2012).

Some lathyrane-type diterpenoids, including compounds **52**–**54**, were isolated from *Euphorbia lagascae* (Table 2). Their effects on the reversal of MDR were examined on mouse lymphoma cells. Among them compound **53** displayed the highest inhibition of Rh123 efflux of human *ABCB1* gene transfected mouse lymphoma cells (FAR = 102.1 at 40 μg/ml) (Duarte et al., 2006). Duarte et al. also isolated compounds **55**–**57** from *Euphorbia lagascae* (Table 1) and evaluated their biological activity against MDR on mouse lymphoma cells. Compounds **55** and **57** showed very strong activity compared with the positive control verapamil (FAR = 110.4 and 216.8 at 4 μg/ml, respectively) (Duarte et al., 2007).

Compounds **58** and **59** were isolated from *Euphorbia piscatoria* (Table 2). Their biological activity against MDR was evaluated through a drug combination assay in the L5178Y mouse T lymphoma cell line transfected with the human *ABCB1* gene. They were able to synergistically enhance the antiproliferative activity of ADR. Most notably, they were further investigated if this synergistic effect could be relevant to the inhibition of ABCB1, using the Rh123 efflux assay, which was negative. These results indicated that these compounds had the reversal effect of MDR independent from ABCB1 by targeting other cellular pathways that are responsible for MDR (Reis et al., 2014).

Twenty new ingol diterpenoids, which are a subgroup of lathyrane diterpenoids, were isolated from *Euphorbia marginata*. All compounds were tested for their biological activity against MDR on ABCB1-dependent MDR cancer cell line HepG2/ADR, and compounds **60**–**62** were identified as potent MDR modulators (Table 19). They enhanced the efficacy of antitumor drug ADR to about 20 folds at 5 μΜ (Zhang et al., 2020).

By SAR studies, it was concluded that the presence of an aromatic component on the lathyrane scaffold significantly improved the inhibition of Rh123 efflux (Reis et al., 2020).

### Clerodanes

Some diterpenoids were extracted from *Sindora sumatrana* (Fabaceae) and their effects on ABCB1 in a ADR-resistant human breast cancer cell line were investigated (Table 20). Among them, compound **63** inhibited the function of ABCB1, which increased the accumulation of ADR by more than four times. Research on SAR indicated that the furan ring had an important effect on its inhibitory activity (Jung et al., 2010).

The ability to modulate the MDR by compounds **64**–**66** was assayed in the MCF-7 cancer cell line (Table 3). The results showed that compounds **64**–**66** were less active as MDR modulators than teotihuacanin, a rearranged clerodane diterpene with potent modulatory activity of MDR in the MCF-7 cancer cell line resistant to vinblastine (Bautista et al., 2016).

**TABLE 3:** Clerodanes. 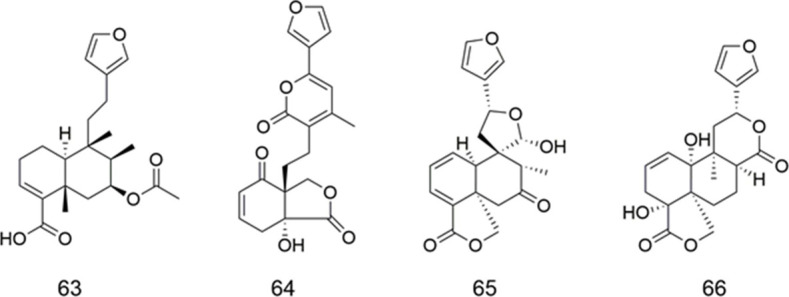

Compound	Name	Plant	Ref
**63**	(+)-7β-acetoxy-15,16-epoxycleroda-3,13 (16),14-trien-18-oic acid	*S. sumatrana*	Jung et al. (2010)
**64**	amarissinin A	*S. amarissima*	Bautista et al. (2016)
**65**	amarissinin B	*S. amarissima*	Bautista et al. (2016)
**66**	amarissinin C	*S. amarissima*	Bautista et al. (2016)

### Pimaranes

Compounds **67** and **68** were isolated from *Ephemerantha lonchophylla* (Orchidaceae) (Table 4)*.* Both of them showed the capability to sensitize B16/hMDR-1 cells with MDR phenotype to the toxicity of the anticancer drug ADR However, both compounds were very weak inhibitors of ABCB1, with ED_50_ values of 193 and 195 µM for compounds **67** and **68**, respectively. In contrast, the ED_50_ of verapamil, an effective ABCB1 inhibitor, was approximately 3 µM (Ma et al., 1998).

**TABLE 4:** Pimaranes. 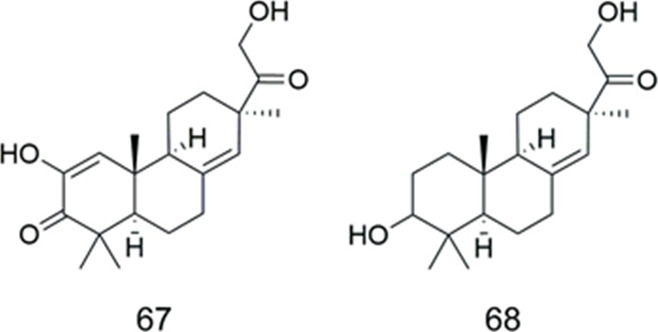

Compound	Name	Plant	Ref
**67**	lonchophylloid A	*E.lonchophylla*	Ma et al. (1998)
**68**	lonchophylloid B	*E.lonchophylla*	Ma et al. (1998)

### Ingenanes

Compound **69** and compound **70** were isolated from *Euphorbia kansui* (Table 5). Compound **70** showed significant MDR reversal activity and compound **69** exhibited moderate MDR reversal activity in HepG-2/ADR cells (RF = 186.4 at 3.87 μM and 57.4 at 12.6 μM, respectively) (Wang S et al., 2021).

**TABLE 5:** Ingenanes. 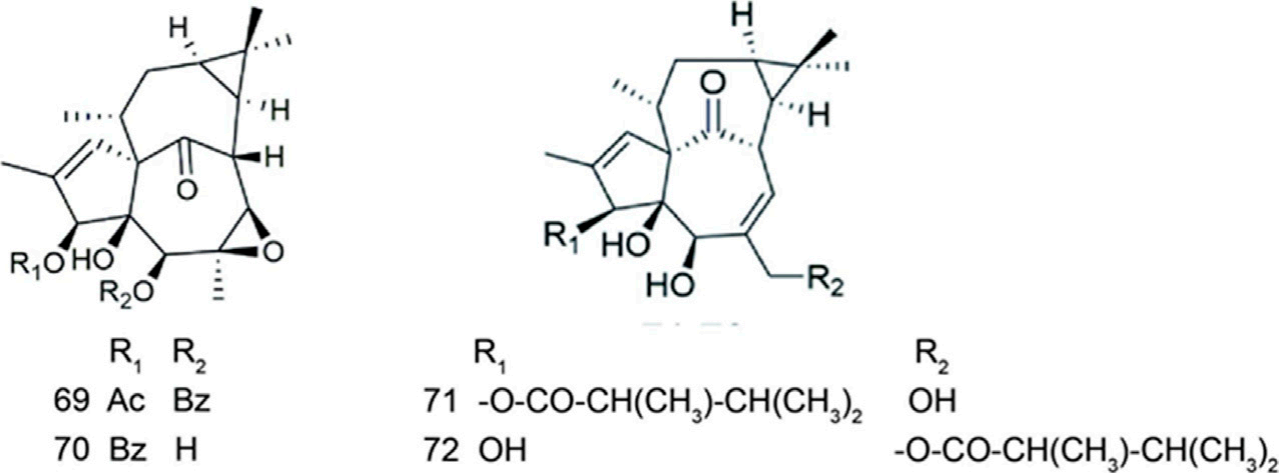

Compound	Name	Plant	Ref
**69**	euphorksol A	*E. kansui*	Wang S et al. (2021)
**70**	6β,7β-epoxy- 3β,4β,5β-trihydroxyl-20-deoxyingenol	*E. kansui*	Wang S et al. (2021)
**71**	kansuininol A	*E. kansui*	Chen et al. (2021)
**72**	kansuininol B	*E. kansui*	Chen et al. (2021)

Two undescribed compounds (**71** and **72**) were isolated from *Euphorbia kansui* (Table 5). The results showed that compounds **71** and **72** were potent low-cytotoxic MDR modulators with greater ability to reverse MDR than verapamil on ADR resistant human breast adenocarcinoma cell line MCF-7/ADR (RF = 21.5 and 18.8 at 5 μM, respectively) (Chen et al., 2021).

### Segetane

Compound **73** was isolated from *Euphorbia taurinensis *(Table 6)*.* It showed significant MDR modulating effect (FAR = 44.44 at 20 µM) in the L5178 mouse lymphoma cell line (Redei et al., 2018).

**TABLE 6:** Segetanes. 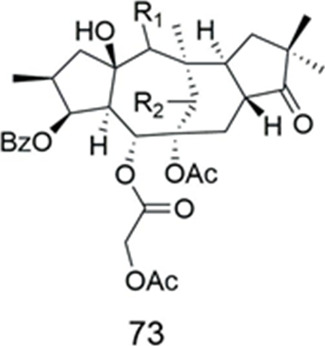

Compound	Name	Plant	Ref
**73**	6,14-Diacetoxy-5-(2-acetoxyacetoxy)-3-benzoyloxy-15-hydroxy-9-oxo-segetane	*E. taurinensis*	Redei et al. (2018)

### Briaranes

Compounds **74**–**76** were isolated from the gorgonian *Briareum excavatum* (Briareidae) (Table 7). Compound **74** completely reversed the resistance to colchicine in KB-C2 cells and showed weak cytotoxicity at 10 μg/ml. From the SAR study, each of the double bond at C-11 and 2,3 and 14-acetoxyl groups in compound **74** were found to be essential to the MDR reversing activity (Aoki et al., 2001).

**TABLE 7:** Briaranes. 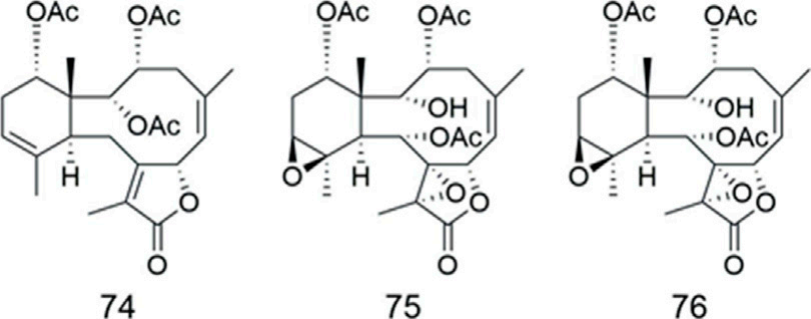

Compound	Name	Source	Ref
**74**	brianthein A	*B. excavatum*	Aoki et al. (2001)
**75**	brianthein B	*B. excavatum*	Aoki et al. (2001)
**76**	brianthein C	*B. excavatum*	Aoki et al. (2001)

### Jatropholane

Compound **77** was isolated from *Euphorbia macrorrhiza* (Table 8). It was tested for cytotoxicity by MTT assay in the human oral epidermoid carcinoma (KB) cell line, using its navelbine-selected ABCB1 overexpressing (KBv200) cell line as experimental model. It was found to exhibit weak cytotoxicity against both KB and resistant KBv200 sublines. Compound **77** was tested along with the classic chemotherapeutic drug navelbine for modulability of MDR against a KBv200 cell line that overexpresses ABCB1 in which verapamil, a well-known chemosensitizer, was used as the positive control. The IC_50_ values for navelbine in combination with compound **77** decreased (from 2.14 to 0.48 μM), suggesting that compound **77** had MDR reversal potential. However, compound **77** was much less active in the MDR reversal assay (RF = 4.47 at 10 μM), compared to that of the positive control (RF = 43.63 at 10 μM) (Gao et al., 2016).

**TABLE 8:** Jatropholane. 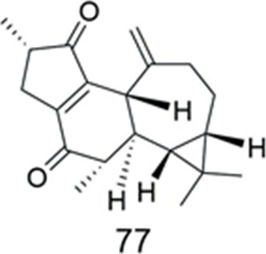

Compound	Name	Plant	Ref
**77**	sikkimenoid A	*E. macrorrhiza*	Gao et al. (2016)

### Pseudolaric Acid

Compound **78** was isolated from *Pseudolarix amabilis* (Pinaceae) (Table 9). A study was conducted on the efficacy of compound **78** toward MDR phenotypes in a ABCB1-overexpressing cell line. The results showed that compound **78** circumvented MDR induced by ABCB1 overexpression (Wong et al., 2005). Sun et al. carried a study on the underlying molecular mechanisms involved in the MDR reversing activity of compound **78**. It was demonstrated that compound **78** (5, 10, and 20 μmol/L) alone or in combination with ADR could inhibit protein expression levels of ABCB1, and reversed MDR of gastric neoplasm to anticancer drugs by downregulating the Cox-2/PKC-α/ABCB1/mdr1 signaling pathway in human gastric cancer SGC7901/ADR Cells (Sun and Li, 2014). Other studies have reached similar conclusions (Yu et al., 2015).

**TABLE 9:** Pseudolaric acid. 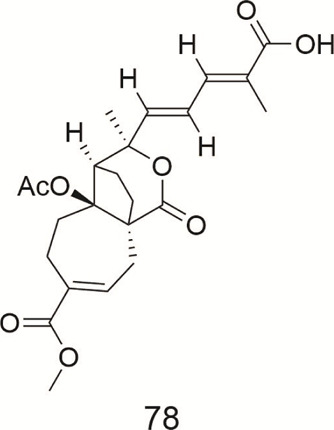

Compound	Name	Plant	Ref
**78**	pseudolaric acid B	*P. kaempferi*	Wong et al. (2005)
			Sun and Li, (2014)
			Yu et al. (2015)

### Taxanes

Compounds **79–81** (Table 10) were isolated from Japanese yew *Taxus cuspidata* (Taxaceae). In all compounds but one, these taxoids (10 μg/ml) increased cellular accumulation of vincristine in multidrug-resistant 2780AD cells (Kobayashi et al., 1994). Some taxoids were isolated from Japanese yew *Taxus cuspidata*, among which compounds **79–80, 82–86** increased cellular accumulation of vincristine in multidrug-resistant human ovarian cancer 2780AD cells as potent as verapamil (Kobayashi et al., 1997). Regardless natural or designed, some taxoids may be good regulators of MDR in cancer chemotherapy (Kobayashi et al., 1998). The research also showed that compound **80** interacted directly with ABCB1 and overcome MDR *in vivo*, like verapamil (Kobayashi et al., 2010). The 6/8/6-membered ring system of some taxanes took commonly “cage”-like backbone structures, which might be important for their effective affinity to ABCB1 (Kobayashi and Shigemori, 2002).

**TABLE 10:** Taxanes. 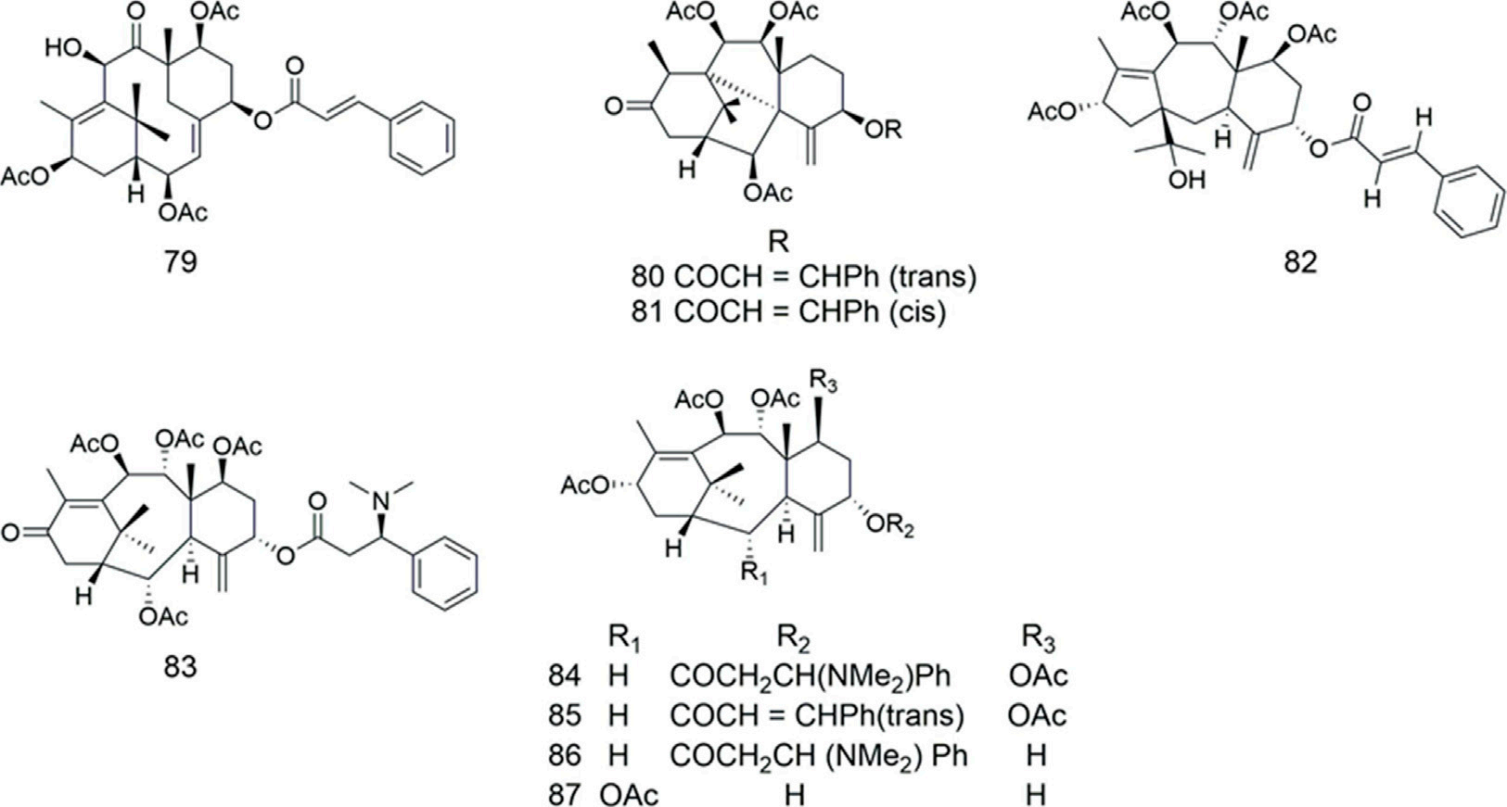

Compound	Name	Plant	Ref
**79**	taxuspine B	*T. cuspidata*	Kobayashi et al. (1994)
			Kobayashi et al. (1997)
**80**	taxuspine C	*T. cuspidata*	Kobayashi et al. (1994)
			Kobayashi et al. (1997)
			Kobayashi et al. (1998)
**81**	7H-6a,10-Methano-1H-benz [c]azulene, 2-propenoic acid deriv	*T. cuspidata*	Kobayashi et al. (1994)
**82**	taxuspine J	*T. cuspidata*	Kobayashi et al. (1997)
**83**	taxine II	*T. cuspidata*	Kobayashi et al. (1997)
**84**	2-desacetoxyaustrospicatine	*T. cuspidata*;	Kobayashi et al. (1994)
		*T. mairei*	Kobayashi et al. (1997)
**85**	2-desacetoxytaxinine J	*T. cuspidata*	Kobayashi et al. (1997)
**86**	7,2′-didesacetoxyaustrospicatine	*T. cuspidata*	Kobayashi et al. (1997)
**87**	2-decinnamoyltaxinine J	*T. cuspidata*	Kobayashi et al. (1997)

### Euphoractine

Compound **88** was isolated from *Euphorbia soongarica *(Table 11)*.* It was tested for MDR reversal activity using the Rh123 accumulation assay in KBv200 cell lines. The results showed that its activity against MDR was lower than that of verapamil (FAR = 0.63 at 10 μM), which was inactive (Gao and Aisa, 2017).

**TABLE 11:** Euphoractine. 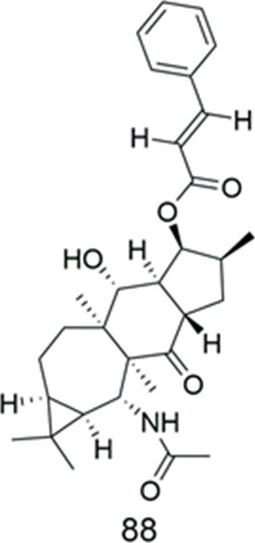

Compound	Name	Plant	Ref
**88**	sooneuphoramine	*E.soongarica*	Gao and Asia, (2017)

## Conclusions

In summary, many diterpenic structures showed MDR-reversal potential*.* Most of the diterpenoids with significant activity against MDR were jatrophane and lathyrane macrocyclic diterpenes isolated from *Euphorbia* species. Aiming at optimizing the structures of diterpenes for reversing MDR, some researchers have prepared hemi-synthetic derivatives, allowing SAR studies.

Inhibiting of ABCB1 function or expression can reverse ABCB1-mediated MDR in cancer cells, which can increase the efficacy of chemotherapy. For the compounds mentioned in this review, inhibiting ABCB1 function was the most common mechanism. For example, compound **10** exhibited superior MDR reversal effect in MCF-7/ADR cells due to the enhancement of ATPase. In addition, compound **10** did not downregulate expression of ABCB1 and mRNA levels in MCF-7/ADR cells (Yang et al., 2021). The most common drug-resistant cell lines involved in this review are HepG2/ADR and MCF-7/ADR cell lines. Most of the active diterpenes were lipophilic compounds, thus corroborating previous studies that defined effective ABCB1 modulator candidates should have a log *p* value of 2.92 or higher (Wang et al., 2003). SAR studies on macrocyclic diterpenes emphasized the importance of an aromatic moiety for ABCB1 binding, through electronic and steric interactions (Reis et al., 2013).

However, most of the works reported focused on cell experiments *in vitro*, and only few studies moved forward to the experiment *in vivo* and showed a certain effect (Zhu et al., 2016; Fang et al., 2018). Thus, further in-depth *in vivo* studies of these compounds are urgently needed (Dong et al., 2020). Moreover, pharmacokinetics studies and the evaluation of the potential toxicity of compounds should also be carried out. Some researchers synthesized a series of derivatives and studied their SAR on the basis of retaining the pharmacodynamic groups of diterpenes (Wang et al., 2020).

Also, the mechanisms of their action were studied by cell biology and molecular biotechnology, which showed that they were being further developed. It is expected to obtain compounds with strong activity and good water solubility, and further confirm their pharmacological activity in *in vivo* experiments. It would also important to assess the ability of these compounds to modulate other ABC transporters involved in MDR, namely ABCC1 and ABCG2.

According to the different structures, the MDR activities of some diterpenoids were described based on their structures. Most of the diterpenoids with good activity against MDR, such as jatrophanes, ingenanes and lathyranes, were isolated from *Euphorbia* species. Some compounds from other species, such as *Pseudolarix*, *Taxus*, *Briareum*, *Sindora* species and so on, also have shown certain reversal activity. Therefore, there is great hope to find more lead compounds from those species, which can reverse MDR and enhance the sensitivity of cancer cells to chemotherapeutic drugs. Overall, diterpenoids with good activity against MDR and low toxicity from natural sources could be developed into lead compounds of new drugs. The structures of diterpenes have important guiding significance for further searching for new drugs to reverse tumor MDR.

## Abbreviations

ABCB1, ATP-binding cassette subfamily B member 1; ABCC1, multidrug resistance-associated protein 1; ABCC2, multidrug resistance-associated protein 2; ABCG2, breast cancer resistance protein; FAR, fluorescence activity ratio; RF, reversal fold; MDR, multidrug resistance; SAR, structure activity relationship; Rh123, Rhodamine 123; ADR, adriamycin; VCR, vincristine; TAX, paclitaxel; NF-κB; nuclear factor κB; MAPK, mitogen-activated protein kinases; PI3K-PKB, phosphoinositide-3-kinase-protein kinase B; IC_50_, half maximal inhibitory concentration; EC_50_, half maximal effective concentration; MTT, 3-(4, 5-dimethylthiazol-2-yl)-2, 5-diphenyltetrazolium bromide.
